# Metabolic adaptations of extremophiles and their applications in environmental biotechnology

**DOI:** 10.1007/s11274-026-05124-4

**Published:** 2026-07-10

**Authors:** Vladimíra Krempaská, Michaela Murgašová, Matej Vesteg

**Affiliations:** Department of Biology and Environmental Studies, Faculty of Natural Sciences, Matej Bel University, Tajovského 40, Banská Bystrica, 974 01 Slovakia

**Keywords:** Extremophiles, Environmental biotechnology, Bioremediation, Bioleaching, Extremozymes, Plastic biodegradation, Pollutant degradation

## Abstract

Extremophiles are microorganisms capable of surviving and thriving under extreme environmental conditions, such as high or low temperatures, extreme pH, elevated pressure or high salinity. These organisms have evolved specialized physiological and biochemical adaptations that enable them to metabolize toxic compounds that are typically lethal to microorganisms living in moderate habitats. Owing to their unique metabolic and enzymatic capabilities, extremophiles are promising candidates for degradation of hazardous pollutants in environments where other microorganisms often exhibit limited activity. This review focuses on the metabolic adaptations and current applications of extremophiles in sustainable environmental biotechnology. In contrast to other reviews focusing primarily on extremozymes or individual remediation strategies, we integrate current knowledge about extremophile physiology with emerging applications fo the removal of plastics, toxic metals (TMs) and petroleum-derived compounds, and we describe the molecular mechanisms employed by extremophiles in these processes. We also discuss current limitations and future research directions in biotechnologies based on extremophiles.

## Introduction

Extremophilic organisms (extremophiles) represent a fascinating group of microorganisms capable of surviving under conditions that would be inhospitable or even lethal to most other forms of life. Their natural habitats include hydrothermal vents with extremely high temperatures and pressure, frozen Arctic and Antarctic environments, hypersaline lakes with high salt concentrations, acidic mine drainages enriched in toxic metals, as well as environments exposed to intense ionizing radiation (Obruča et al. 2018). Extremophiles are classified according to the environmental stressors they tolerate. These are extreme temperatures (thermophiles and psychrophiles), extreme pH (acidophiles and alkaliphiles), various factors increasing osmotic pressure (osmophiles, halophiles and xerophiles), extreme pressure (piezophiles), high concentration of toxic metals (metallophiles) or high radiation (radiophiles) (Wang et al. 2024b). Various examples of extremophiles are listed in Table 1. Many of these microorganisms are polyextremophiles, meaning that they can withstand multiple extreme factors simultaneously, which makes them particularly valuable for biotechnological applications, especially for bioremediation (Dopson et al. 2023).

**Table 1:** Examples of extremophiles

Category	Type of extremophiles	Examples (genera)	References
Temperature	Thermophiles (40–110 °C)	Archaea: *Pyrococcus*, *Thermococcus*, *Methanococcus*, *Sulfolobus*, *Metallosphaera*	Maheshwari et al. 2000; Straub et al. 2018; Finore et al. 2023
		Bacteria: *Aquifex*, *Hydrogenobacter*, *Bacillus*, *Geobacillus*, *Thermus*, *Thermotoga*	
		Eukarya: *Canariomyces*, *Chaetomium*, *Coonemeria*, *Corynascus*	
	Psychrophiles (–20–20 °C)	Bacteria: *Alteromonas*, *Shewanella*, *Psychrobacter*, *Pseudoalteromonas*, *Arthrobacter*,* Colwellia*, *Gelidibacter*, *Pseudomonas*, *Psychroflexus*	Franzmann et al. 1997; Moyer and Morita 2007; De Maayer et al. 2014
Acidity	Alkaliphiles (high pH > 8.5)	Archaea: *Halalkalicoccus*, *Halobiforma*, *Halorubrum*, *Natrialba*, *Natronococcus*, *Natronorubrum*	Cárdenas et al. 2010; Bowers and Wiegel 2011; Sarethy et al. 2011; Navarro et al. 2013; Dhakar et al. 2014; Dopson and Holmes 2014
		Bacteria: *Bacillus*, *Halomonas*, *Pseudomonas*	
		Eukarya: *Cladosporium*, *Fusarium*, *Penicillium*, *Sodiomyces*, *Thielavia*	
	Acidophiles (low pH < 5)	Archaea: *Sulfolobus*, *Ferroplasma*, *Picrophilus*, *Thermoplasma*	Dhakar et al. 2014; Abbamondi et al. 2019; Tripathi et al. 2021
		Bacteria: *Acidithiobacillus*, *Acidiphilium*, *Bacillus*, *Acidothrix*	
		Eukarya: *Scytalidium*, *Aspergillus*, *Cryptococcus*, *Phialophora*, *Trichoderma*, *Trichosporon*	
Pressure	Piezophiles (up to 340 MPa)	Archaea: *Thermococcus*,* Pyrococcus*	Zhang et al. 2015; Sarma et al. 2023
		Bacteria: *Colwellia*, *Shewanella*, *Moritella*	
Osmotic pressure	Halophiles (high salinity, 3–30% NaCl)	Archaea: *Haloferax*,* Halococcus*,* Haloquadratum*,* Halobacterium*	Oren 2005; Stan-Lotter and Fendrihan 2015; Corral et al. 2019; Alvares and Furtado 2021
		Bacteria: *Salinispora*, *Halomonas*, *Vibrio*, *Nocardiopsis*, *Bacillus*, *Streptomyces*	
		Eukarya: genus *Dunaliella*	
	Osmophiles (high sugar content)	Eukarya: genus *Zygosaccharomyces*	Wang et al. 2024a
	Xerophiles (dry environments)	Eukarya: genera *Zygosaccharomyces*, *Debaryomyces*	Rao et al. 2022
Toxic metals	Metallophiles	Archaea: *Ferroplasma*, *Acidiplasma*, *Sulfolobus*, *Metallosphaera*, *Acidianus*	Dopson and Holmes 2014; Manesh et al. 2024
		Bacteria: genera *Acidithiobacillus*, *Leptospirillum*, *Alicyclobacillus*, *Acidiphilium*, *Acidimicrobium*, *Ferrimicrobium*, *Sulfobacillus*	

Extremophiles are found across all three domains of life (Archaea, Bacteria, and Eukarya), while extremophilic archaea and bacteria are the most abundant representatives. Their physiological diversity is quite high, including aerobic and anaerobic microorganisms, chemotrophs and phototrophs, lithotrophs and organotrophs, and autotrophs and heterotrophs (Barrie Johnson and Hallberg et al. 2009). The ability to survive in harsh environments is enabled by unique adaptive mechanisms. These adaptations include specialized cell wall structures, enhanced intracellular protein stability against denaturation, and the ability to maintain genetic material integrity under extreme conditions (Durvasula and Rao 2018). Particularly remarkable are their unique enzymes (extremozymes), which remain catalytically active under conditions inhibiting the activity of conventional biocatalysts (Sikdar et al. 2024).

The exceptional stability and activity of extremozymes make extremophiles highly attractive for biotechnological applications. Extremozymes are increasingly explored for industrial, environmental and pharmaceutical purposes, particularly in processes involving extreme temperatures, pH or salinity (Raddadi et al. 2015). Their use is especially widespread in the food, pharmaceutical, detergent and environmental industries (Mutlu-Ingok et al. 2022; Sepe et al. 2025). With the growing emphasis on the bioeconomy and sustainable industrial production, biocatalysis based on extremophilic microorganisms has emerged as a promising alternative to many chemical processes (Mesbah 2022).

Extremophiles have also become important model organisms in next-generation industrial biotechnology (NGIB), which aims to develop robust microbial production systems capable of operating under non-sterile or extreme cultivation conditions (Wang et al. 2024b). Due to their ability to thrive under harsh environmental conditions, extremophiles may reduce contamination by mesophilic microorganisms, decrease sterilization requirements and lower operational costs associated with industrial bioprocesses (Obruča et al. 2022). For example, thermophilic fermentation processes can operate at temperatures above 50 °C, which are lethal to mesophiles (OHair et al. 2021). Nevertheless, the economic feasibility and scalability of extremophile-based industrial systems remain insufficiently evaluated. Further studies are required to assess their long-term efficiency and industrial competitiveness in comparison to commonly used mesophilic platforms.

This review summarizes current knowledge about metabolic adaptations of extremophiles and their applications in environmental biotechnology, with particular emphasis on biodegradation of plastics, petroleum-derived pollutants and toxic metals. In contrast to previous reviews focused primarily on extremozymes or specific remediation strategies, we integrate extremophile physiology, biodegradation mechanisms and industrial applications under extreme environmental conditions. Furthermore, we critically discuss current limitations associated with extremophile-based biotechnologies, including cultivation challenges, scalability, ecological risks and industrial feasibility. Finally, future perspectives involving NGIB, synthetic biology and omics-based approaches are highlighted as promising directions for the development of sustainable bioremediation technologies.

## Molecular adaptations of extremophiles

From a biochemical perspective, extremophiles exhibit highly specialized adaptations that allow them to survive under conditions that would typically denature proteins, disrupt cell membranes or damage genetic material. These adaptations include unique modifications of cellular structures, enhanced stability of intracellular proteins and nucleic acids, and the ability to maintain homeostasis in habitats with extreme pH, temperature, pressure or salinity (Gallo and Aulitto 2024). Although the molecular basis of their stability is in most cases not yet fully understood, some studies suggest that hydrogen and covalent bonds, amino acid composition (Zeldovich et al. 2007), G + C content in DNA and specific rRNA folding patterns play crucial roles (Satapathy et al. 2010; Kumar et al. 2019). By understanding these molecular adaptations, researchers can develop novel biotechnological solutions, such as sustainable industrial processes and strategies for the decontamination of polluted areas (Nadaroglu and Polat 2022). In the following sections, we have focused on the specific adaptations of different extremophile groups and their mechanisms for surviving in hostile environments.

### Extreme temperatures: adaptations to high and low temperatures

Mesophiles, the most extensively studied organisms, grow optimally at 25–40 °C (Vavitsas et al. 2022). Thermophiles require temperatures exceeding 40 °C, while hyperthermophiles can grow optimally from 80 to 110 °C (Wang et al. 2024b). These heat-loving microbes have developed specialized adaptations to maintain cellular integrity and prevent biomolecular denaturation. Notable archaeal hyperthermophiles which thrive at temperatures above 60 °C include genera such as *Pyrococcus* (class Thermococci) with optimal growth at around 100 °C (Fiala and Stetter 1986), *Thermococcus* (class Thermococci) with growth temperatures at 60–105 °C (Zhang et al. 2012), and *Sulfolobus* (class Thermoprotei) with optimal growth at 75–80 °C (Beeckmans 2009; Atomi et al. 2011). There are also known bacterial thermophiles such as genera *Aquifex* (class Aquificia) with optimal growth at around 85–90 °C (Laemthong et al. 2022) and *Thermotoga* (class Thermotogae), which flourishes at temperatures exceeding 65 °C, sometimes even reaching 80 °C (Laemthong et al. 2022). Other bacterial representatives, such as genera *Anoxybacillus*, *Brevibacillus* and *Geobacillus* (all belonging to class Bacilli), grow at moderate thermophilic ranges from 41 to 50 °C. These bacteria are widely utilized in industrial applications, including starch hydrolysis, paper bleaching and protease production for food processing (Goswami et al. 2024). A particularly well-known thermophile, bacterium *Thermus aquaticus*, with optimal growth temperature 65–70 °C but surviving at 50–80 °C, has revolutionized molecular biology through the discovery of Taq polymerase, an enzyme still most commonly used for polymerase chain reaction (PCR) (Arbab et al. 2022).

Thermophiles exhibit several key adaptations that ensure the stability of their biomolecules under extremely hot conditions. Their DNA is rich in G + C base pairs, enhancing thermal stability, while their proteins contain a higher number of disulfide bonds to maintain structural integrity (Brininger et al. 2018; Mohanty et al. 2022). Additionally, thermophiles possess structurally optimized rRNA and tRNA molecules with enhanced thermal stability, which is essential for maintaining ribosome integrity and efficient protein translation at high temperatures (Jegousse et al. 2017). Their lipid membranes are also more rigid, containing unique lipids that increase heat resistance and prevent membrane disruption (Stetter 2006). Beyond structural adaptations, thermophiles demonstrate exceptional metabolic versatility. They can utilize a wide range of substrates, including polysaccharides (starch, cellulose, xylan, and pectin), peptides, hydrocarbons, and inorganic compounds like molecular hydrogen and elemental sulfur (Zeldes et al. 2015). Although their metabolic pathways share similarities with those of mesophiles, thermophiles exhibit specialized enzymatic adaptations to sustain efficient biochemical reactions at elevated temperatures. This metabolic flexibility enhances their potential for industrial bioprocesses, offering sustainable alternatives for energy-efficient bioconversions (Vavitsas et al. 2022). The ability of thermophiles to tolerate extreme heat has profound implications for biotechnology, particularly in enzyme and biofuel production, and waste treatment.

Psychrophiles, on the other hand, can survive and thrive at low temperatures, ranging from − 20 to 20 °C, with their optimal growth temperature typically around 15 °C or lower (Goswami et al. 2024). They are found in a wide range of cold habitats, including polar regions, high-altitude soils, deep-sea ecosystems and glaciers, which together cover more than 70% of the Earth’s surface. Typical bacterial representatives include members of the class Gammaproteobacteria, particularly species belonging to the genera *Pseudomonas*, *Psychrobacter*, *Pseudoalteromonas*, and *Colwellia* (Goswami et al. 2024). Survival in cold environments requires overcoming several thermodynamic challenges, such as reduced molecular mobility resulting from low entropy and enthalpy. To achieve this, they have developed specific adaptive mechanisms that allow them to maintain biological functions. One of the key adaptive mechanisms of psychrophiles is the production of antifreeze biomolecules, such as glycoproteins and polysaccharides, which prevent the formation of ice crystals and protect cellular structures from damage (Raddadi et al. 2015). These include extracellular polysaccharides like alginate and mannitol, which act as cryoprotectants in biofilms and help to stabilize the cellular environment (Białkowska et al. 2020). In addition, psychrophiles possess proteins with a higher proportion of smaller amino acids and a lower content of proline, which limits the rotation of protein chains (Feller and Gerday 2003). For example, psychrophilic alpha-amylase contains only 13 proline residues, while its mesophilic and thermophilic counterparts contain 19 and 25, respectively (Feller 2018). The lower proline content increases protein flexibility, ensuring their functionality in cold environments.

Psychrophilic proteases, lipases, and cellulases are highly catalytically active and stable at low temperatures, making them extremely valuable for industrial applications. They are utilized in biotechnology, including industrial detergent production, food processing, bioremediation and molecular biology (Arbab et al. 2022). Their ability to function at low temperatures reduces energy costs and provides an environmentally friendly alternative to enzymes from mesophiles.

### pH Extremes: adaptations to acidic and alkaline environments

Microorganisms that grow under extremely acidic or alkaline conditions are classified as acidophiles and alkaliphiles, respectively. Acidophiles prefer environments with a pH below 5, while alkaliphiles are found in environments with a pH over 8.5 (Moussa and Khalil 2022). Typical habitats for acidophiles include acidic hot springs, acidic mines and volcanic areas, whereas alkaliphiles are commonly found in alkaline lakes, groundwater and gold mines (Misra et al. 2021). In general, many acidophiles are also thermophilic, possessing similar adaptation mechanisms to those mentioned above (Reed et al. 2013). Acidophiles possess membranes with reduced permeability and they also employ mechanisms for pumping protons out of the cell to ensure stable neutral intracellular pH levels (Aliyu et al. 2024). In contrast, alkaliphiles are adapted to high pH environments by relying primarily on Na⁺/H⁺ antiporters, which expel Na^+^ out of the cell while importing protons (Misra et al. 2021).

The example of one of the most acidic environments is the Iron Mountain in Shasta County, California (Johnson 2012). Microorganisms thriving there include bacterial species such as *Acidithiobacillus ferrooxidans* (class Acidithiobacillia) and *Leptospirillum ferrooxidans* (class Nitrospira) (Singh et al. 2012). Archaeal representatives include genera *Acidianus*, *Metallosphaera*, *Stygiolobus* and *Sulfolobus* (all belonging to class Thermoprotei), as well as *Thermoplasma* and *Picrophilus* (both belonging to class Thermoplasmata) (Sharma et al. 2012). On the other hand, alkaliphilic bacteria, such as *Mesorhizobium ciceri* and *Mesorhizobium muleiense* (class Alphaproteobacteria), are often found in alkaline soils. Additionally, alkaliphilic bacterial species from the genera *Bacillus* (class Bacilli), *Micrococcus* (class Actinomycetes), *Pseudomonas* (class Gammaproteobacteria) and *Streptomyces* (class Actinomycetes) are common representatives of alkaliphiles in various alkaline environments (Goswami et al. 2024).

The study of acidophiles and alkaliphiles has expanded not only our knowledge of microbial adaptation, but also our understanding of their potential in biotechnology. They are increasingly used for bioremediation, bioenergy production and industrial biocatalysis (González et al. 2024). Acidophiles are valuable for metal recovery from ores (biomining), surface polishing of electronic devices (biomachining), and the treatment of acidic mine wastewater (Aliyu et al. 2024). Alkaliphiles, on the other hand, are key producers of alkaline enzymes used as additives in detergents, and they also contribute to the cost-effective production of cyclodextrins, with wide use in the food, chemical and pharmaceutical industries (Horikoshi 1999).

### Adaptations to osmotic stress: halophiles, osmophiles and xerophiles

In environments with reduced water availability due to high solute concentrations or physical desiccation, microorganisms face with osmotic stress that threatens cellular stability and survival. To thrive under these conditions, microorganisms have evolved remarkable strategies to maintain osmotic balance, protect cellular structures and ensure metabolic functionality. These microorganisms include osmophiles adapted to high osmotic pressure caused by elevated concentrations of sugars or salts (Kim et al. 2015), as well as xerophiles adapted to extremely dry habitats with very low water availability (Rao et al. 2022).

Among osmophilic microorganisms, yeasts represent one of the most extensively studied groups because of their ecological and industrial importance (Segal-Kischinevzky et al. 2022). Osmophilic yeasts commonly occur in environments with elevated sugar concentrations, including sugar-rich foods. They are major agents causing spoiling of jams, honey or concentrated fruit juices (Kim et al. 2015). However, the common spoilage agent, *Zygosaccharomyces rouxii* (class Saccharomycetes) can also be applied for certain fermentation processes (Wang et al. 2024a).

Halophiles thrive in environments with high concentrations of NaCl (30–300 g/L), requiring it for their growth. The majority of halophilic representatives are prokaryotes, but eukaryotes, such as green algal species *Dunaliella salina* (class Chlorophyceae), containing orange carotenoid pigments, are also commonly found under hypersaline conditions. The best-known representatives of extreme halophiles are the archaeal members of the class Halobacteria, also known as Haloarchaea (kingdom Methanobacteriati). The recognized diversity of this class, considered to be the archetypal group of halophilic microorganisms, has increased markedly in recent years. Among bacteria, the most extensive assemblage of moderately halophilic, salt-tolerant taxa belongs to the family Halomonadaceae (class Gammaproteobacteria), which now includes more than 160 described species (Oren 2024).

**Fig. 1:**
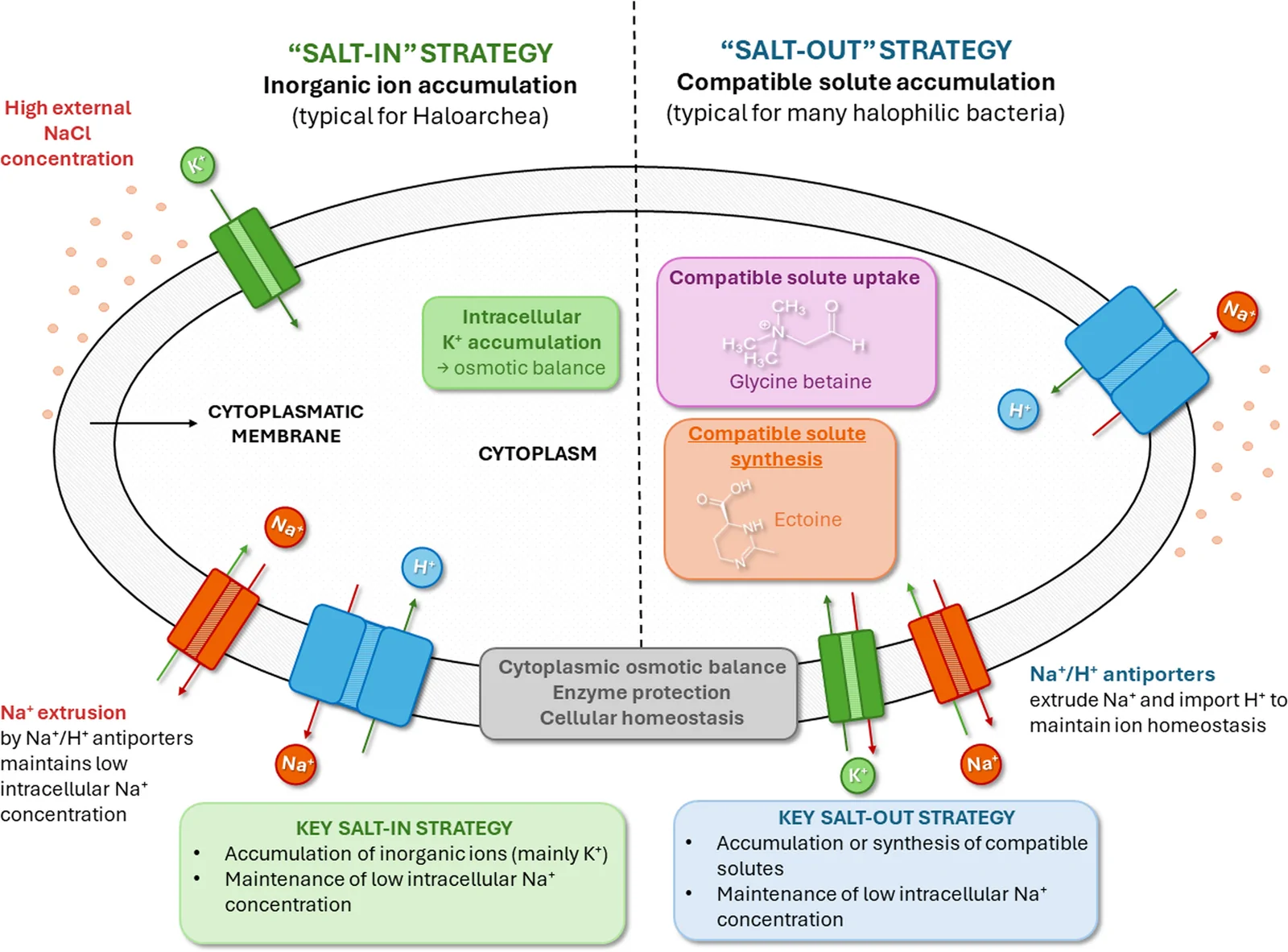
The “salt-in” and “salt-out” strategies of halophiles. The “salt-in” strategy is based on intracellular accumulation of K⁺ ions combined with Na⁺ extrusion through Na⁺/H⁺ antiporters, whereas the “salt-out” strategy relies on synthesis or uptake of compatible solutes (such as ectoine and glycine betaine, respectively) to maintain osmotic balance and cellular stability under hypersaline conditions

Halophiles have developed various strategies to resist the osmotic stress (Ortega et al. 2015). Two main ones are the “salt-in” and “salt-out” strategies (Fig. 1). The first one and the most common osmoadaptation can be observed in most representatives of Haloarchaea. These archaea are capable of accumulating high concentrations of K⁺ in the cytoplasm, while simultaneously exporting Na⁺ cations into the extracellular environment (Oren 2013). They have also evolved an acidic proteome, which ensures that cytoplasmic proteins remain stable and functional under such extreme conditions. However, except for Haloarchaea, this strategy is restricted to relatively few other halophiles. Within the domain Bacteria, this mechanism has been clearly documented for the order Halanaerobiales (class Clostridia) (Martínez et al. 2022) and *Salinibacter ruber* (class Rhodothermia) (Oren 2024).

The second strategy, “synthesis of compatible osmotic solutes”, also known as “salt-out strategy”, is typical for most halophilic bacteria and for certain Haloarchaea. In this case, cells synthesize or import compatible organic solutes, such as ectoine and glycine betaine, respectively, to achieve osmoregulation (Gunde-Cimerman et al. 2018). Other small organic molecules, such as amino acids, sugars or polyols, can also be accumulated in the cytoplasm. Compared with the “salt-in strategy”, this approach costs more energy but is widespread across all three domains of life. For instance, algae of the genus *Dunaliella* accumulate glycerol as their primary compatible solute (Martínez et al. 2022). This strategy also depends on exporting Na⁺ out of the cell via Na⁺/H⁺ antiporters (Bonnaud et al. 2024).

Halophiles also have various biotechnological applications (Yavari-Bafghi and Amoozegar 2025). They have been explored for applications in food fermentation, bioplastic production, wastewater treatment, bioremediation of saline environments and synthesis of bioactive compounds with pharmaceutical potential (Dutta and Bandopadhyay 2022).

Other osmophiles, namely xerophiles, inhabiting arid environments, such as deserts, have also evolved specialized adaptations enabling survival under extreme desiccation conditions (Stevenson et al. 2015). Xerophiles are able not only to tolerate severe water limitation but also radiation, extreme temperatures and pH fluctuations (Rao et al. 2022). Some of them can form highly resistant spores typical for some bacterial genera, such as *Bacillus* and *Clostridium* (Rao et al. 2022). Another strategy is the production of extracellular polysaccharides (EPS) or compatible solutes like glycerol, commonly accumulated by xerophilic yeasts, such as previously mentioned *Z. rouxii* or *Debaryomyces hansenii* (class Pichiomycetes). Other compatible solutes, including ectoine and glycine betaine, offer protection similar to that observed in halophilic microorganisms. Additional adaptations include the synthesis of protective pigments (e.g., carotenoids) and stress-response proteins (LEA proteins), catalases, thioredoxins, and iron-dependent superoxide dismutases (Rao et al. 2022).

### Extreme pressures: adaptations to deep-sea and other high-pressure environments

Extreme hydrostatic pressure represents a unique environmental challenge for microbial life. The pressure on Earth’s surface ranges from atmospheric levels (~ 0.1 MPa) at sea level to over 110 MPa in the deepest parts of subduction zones, such as the Mariana Forearc (Qiu et al. 2024). Despite the physical constraints imposed by such condition, including reduced molecular motion, altered protein-ligand interactions and reduced membrane fluidity, certain microorganisms known as piezophiles (formerly barophiles) have evolved the ability not only to survive but actively proliferate under these extreme pressures (Scheffer and Gieg 2023). Natural habitats of piezophiles include deep-sea sediments, abyssal and hadal trenches, cold seeps and hydrothermal vents. One notable example of archaeal piezophile is *Thermococcus piezophilus* (class Thermococci), a hyperthermophilic archaeon capable of growth at pressures up to 125 MPa (Merino et al. 2019). Some studies suggest that microbial activity may persist even at pressures around 340 MPa in deep lithospheric environments (Merino et al. 2019).

Recent metagenomic and transcriptomic studies have considerably expanded current understanding of the adaptive mechanisms employed by piezophilic microorganisms in deep-sea environments. In particular, Mullis et al. (2023) provided important insights into microbial survival strategies in the Mariana Trench as part of the International Ocean Discovery Program Expedition 366 to the Mariana Convergent Margin. Microbial communities were investigated in samples collected from three serpentinizing seamounts characterized by nutrient-poor and highly alkaline conditions, with pH 10–12.5. Transcriptomic analyses revealed the presence of representatives from all three domains of life, dominated by bacterial orders Burkholderiales (class Betaproteobacteria), Deinococcales (class Deinococci), and class Gammaproteobacteria, as well as filamentous fungi. Functional analyses identified transcripts associated with aerobic methane oxidation and denitrification pathways, while genes involved in membrane and cell wall repair were among the most highly expressed (Mullis et al. 2023).

Piezophiles share several adaptive features with psychrophiles, including reduced protein rigidity allowing functionality at high pressure, lower proline and glycine content favouring increased protein flexibility and there is also a preference for small amino acids, which reduce steric hindrance (Scheffer and Gieg 2023). Since increased pressure tends to rigidify lipid bilayers, piezophiles have membranes enriched in unsaturated and branched fatty acids, maintaining fluidity under compression. Some of them also synthesize pressure-regulated proteins to assist in proper protein folding and repair under stress (Tamby et al. 2023).

An illustrative example of pressure-responsive regulation is the bacterial species *Photobacterium profundum* strain SS9 (class Gammaproteobacteria). It expresses outer membrane proteins OmpH and OmpL in a pressure-dependent manner: OmpH predominates at optimal deep-sea pressures (28 MPa), while OmpL is more abundant at atmospheric pressure (0.1 MPa). These proteins are involved in membrane permeability and may help cells adapt to dynamic pressure changes (Arulazhagan et al. 2017). Interestingly, not all cellular proteins in piezophiles are pressure-regulated. Most metabolic enzymes function normally under different pressures, while only some genes are activated in response to pressure shifts, suggesting a targeted transcriptional response to hydrostatic stress (Arulazhagan et al. 2017).

Piezophiles and their enzymes can also have applications in various processes, such as food sterilization by high pressure or biocatalysis in deep-sea environments. However, cultivating piezophiles under laboratory conditions remains a considerable challenge (Abe and Horikoshi 2001).

### Surviving in toxic zones: adaptations to toxic metals

The presence of toxic metals (TMs) in the environment poses a serious problem, significantly affecting biological processes. Some of the most prevalent TMs found in nature include cadmium (Cd), mercury (Hg), chromium (Cr), lead (Pb), arsenic (As), copper (Cu), nickel (Ni), and zinc (Zn). These TMs and their derivatives can damage cell membranes, modify enzymatic specificity, disrupt DNA structure, and interfere with other cellular components. Therefore, microorganisms have developed various mechanisms to protect themselves against their toxic effects. Many of these microorganisms also belong to the other groups of extremophiles, including thermophiles, psychrophiles, acidophiles, and alkaliphiles (Dopson and Holmes 2014).

**Fig. 2:**
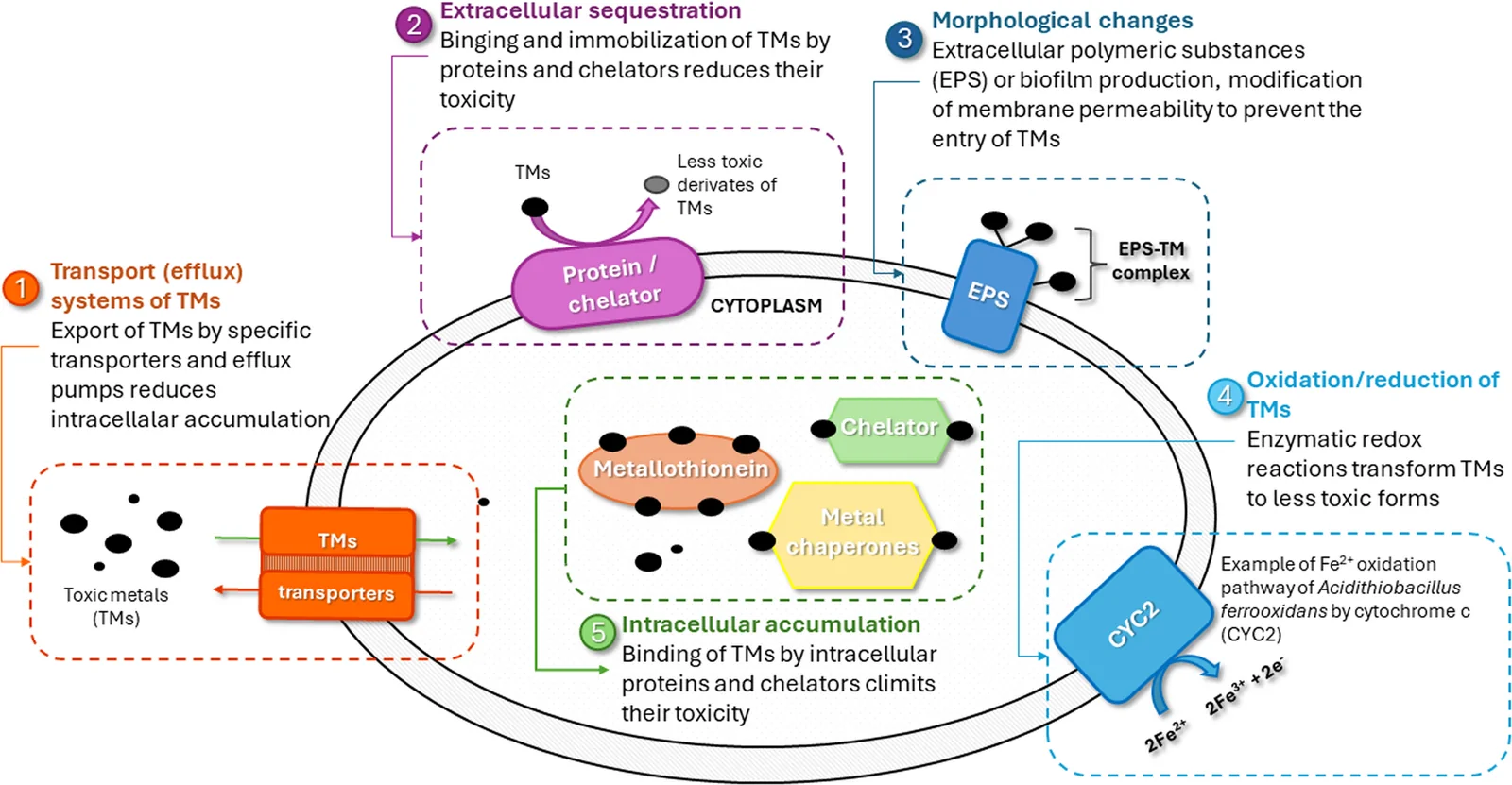
Major mechanisms of TMs resistance and detoxification by extremophiles. These strategies include (1) active transport and efflux of TMs, (2) extracellular sequestration by metal-binding proteins and chelators, (3) morphological changes such as EPS or biofilm production, (4) enzymatic oxidation/reduction reactions mediated by cytochrome c-like protein, and (5) intracellular detoxification by metallothioneins, chelators and metal chaperons

Several mechanisms exist for how microorganisms endure high TMs contamination (Sun et al. 2024). Figure 2 illustrates the main ones. One of them is an active efflux out of the cell by toxic metal transporters. Particularly interesting are also the morphological changes, including modification of membrane permeability, and the production of extracellular polymeric substances (EPS – mainly polysaccharides), which can form complexes with TMs. These complexes enhance the immobilization and precipitation of TMs, thereby reducing their toxicity and ability to get into the cell (Kondakindi et al. 2024). Other crucial mechanisms are extra- and intracellular sequestrations. For extracellular sequestration, microorganisms use proteins and chelators to bind TMs outside the cell, effectively detoxifying them before they enter the cytoplasm. In contrast, intracellular sequestration involves the detoxification of TMs within the cell through protein- or chelator-mediated binding, typically associated with proteins such as metallothioneins and metal chaperones (Nnaji et al. 2024). In some cases, microorganisms are also capable of transforming TMs into less toxic forms through oxidation, reduction, or methylation/demethylation reactions. For example, *Acidithiobacillus ferrooxidans* (class Acidithiobacillia) can oxidize Fe²⁺ via the cytochrome c-like protein CYC2 (Zhan et al. 2019).

Microorganisms often combine several mechanisms to defend against TM’s toxicity. However, the pathways responsible for these adaptations remain an active area of research. Some of the best-known microorganisms resistant to TMs include species from various groups of extremophiles. Among acidophiles, bacterial genera such as *Acidithiobacillus* (class Acidithiobacillia), *Leptospirillum* (class Nitrospira), *Alicyclobacillus* (class Bacilli), *Acidiphilium* (class Alphaproteobacteria), *Acidimicrobium* (class Acidimicrobiia), *Ferrimicrobium* (class Acidimicrobiia), and *Sulfobacillus* (class Sulfobacillia) are notable representatives (Dopson and Holmes 2014). Archaea, including *Ferroplasma* (class Thermoplasmata), *Acidiplasma* (class Thermoplasmata), *Sulfolobus* (class Thermoprotei), *Metallosphaera* (class Thermoprotei), and *Acidianus* (class Thermoprotei), also play key roles in TMs detoxification in acidic environments (Dopson and Holmes 2014; Johnson 2014). In environmental biotechnology, acidophiles are extensively utilized in a process known as biomining, where they facilitate the extraction of metals such as copper and gold through the oxidation of metal sulfides into soluble metal sulfates (Johnson and du Plessis 2015). Although substantial progress has been made in understanding TMs tolerance mechanisms in acidophilic and thermophilic microorganisms, resistance strategies in psychrophiles remain comparatively poorly studied (Chaudhary and Kim 2019). Consequently, numerous microorganisms exhibit polyextremophilic characteristics, enabling simultaneous adaptation to multiple environmental extremes.

### Polyextremophiles: surviving multiple extreme conditions

The study of polyextremophiles surviving multiple extreme conditions does not only deepen our understanding of microbial adaptability but also unlocks new opportunities for biotechnological innovations (Dopson et al. 2023). Polyextremophilic microorganisms include thermo-alkaliphiles and thermo-acidophiles (Angelov and Liebl 2006), as well as haloalkaliphiles (Uma et al. 2020). Another notable example is halo-psychrophile *Halorubrum lacusprofundi*, archaeon that grows at − 1 °C in hypersaline environments (≥ 200 g/L NaCl) and produces enzymes like β-galactosidase with industrial relevance due to its stability under extreme conditions (Laye and Das Sarma 2018). Another group of interest are acidophiles such as bacteria *Acidithiobacillus ferrivorans*, *Acidithiobacillus ferriphilus*, and *Acidithiobacillus ferrooxidans* (all of them belonging to the class Acidithiobacillia). *Acidithiobacillus* spp. grow within a temperature range of 5–37 °C, and a pH range of 1–7.5 (Sriaporn et al. 2021) and are considered promising candidates for environmental biotechnologies, particularly for bioleaching and bioremediation (Dopson et al. 2023). Similarly, *Pseudomonas chlororaphis* demonstrates exceptional physiological flexibility, growing across a wide range of temperatures (5–45 °C), pH (2–14), and salt concentrations (up to 150 g/L), suggesting potential for applications in fluctuating or extreme industrial settings (Jain and Pandey 2016).

Among eukaryotic extremophiles, tardigrades (water bears) are perhaps the most extraordinary. These microscopic animals can survive conditions lethal to almost all other life forms, including temperatures from − 272 °C to 151 °C, pressures exceeding 6000 atm, desiccation, vacuum, and intense radiation (Horikawa 2012). They achieve this through a cryptobiotic state known as a “tun”, which preserves cellular integrity under extreme stress (Goswami et al. 2024). All these examples highlight the untapped potential of polyextremophiles for advanced sustainable biotechnologies.

## Biotechnological applications of extremophiles for bioremediation

**Fig. 3:**
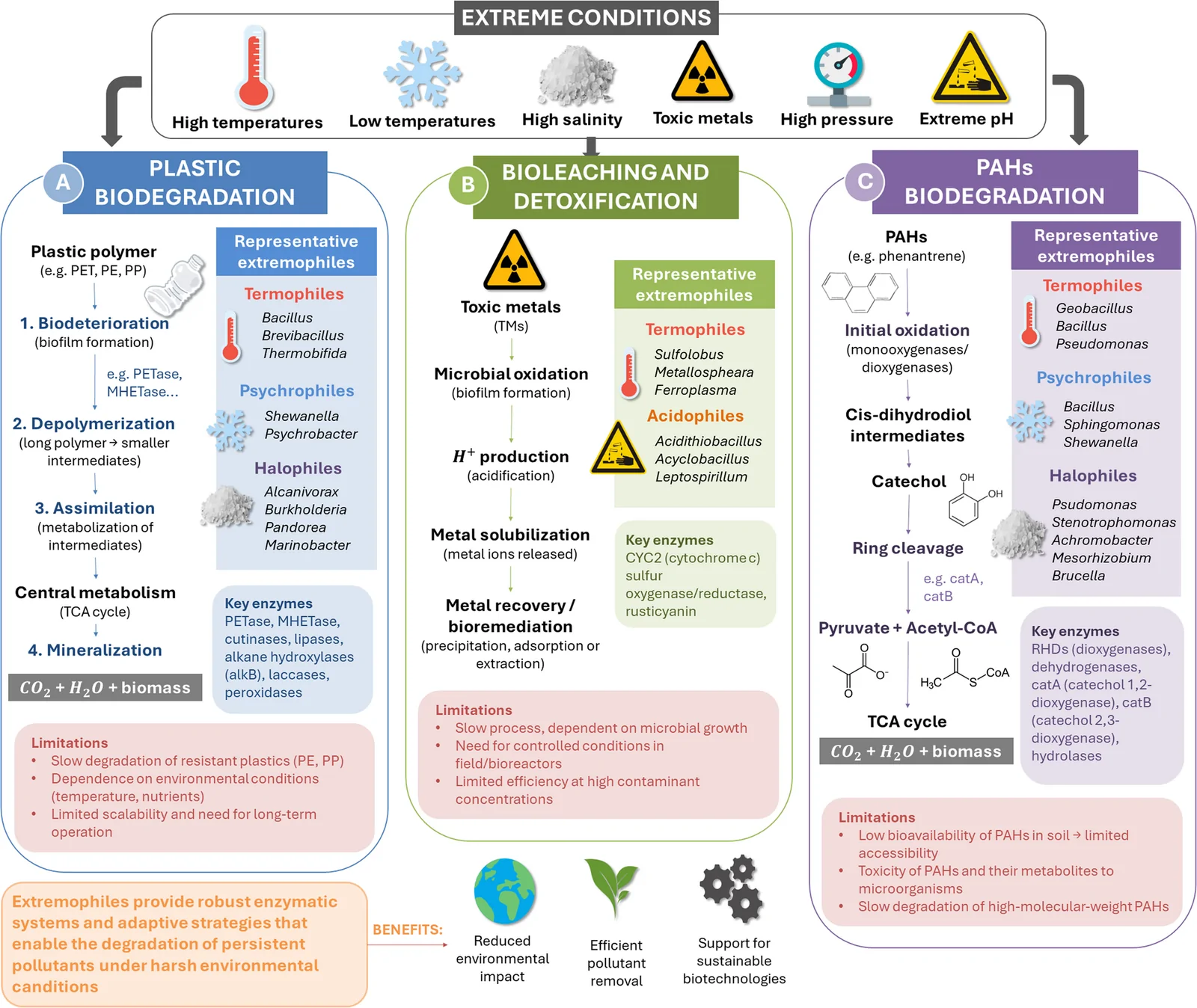
Major mechanisms employed by extremophiles in (**A**) plastic biodegradation, (**B**) metal bioleaching and detoxification, and (**C**) PAHs degradation. Key enzymes/genes, intermediates, representative genera and main limitations are included

Extremophiles have gained significant scientific attention, and their industrial applications have been intensively studied for over 30 years. Extremophilic microorganisms have been applied in food processing (dairy fermentation), agriculture, biofuel production, fermentation technologies, the pharmaceutical industry (antibiotic production), and detergent manufacturing (Rawat et al. 2024). Xylanases, proteases, amylases, pullulanases, pectinases, cellulases, keratinases, lipases, phytases, esterases, peroxidases, and catalases are among the most commonly used extremozymes produced by extremophiles (Haripriyan et al. 2022), exhibiting exceptional stability and activity under extreme conditions (Dumorne et al. 2017). Extremophiles can also be used for bioleaching, liberation or extraction of metals from ore (Adetunji et al. 2023). Oil and ore extraction and processing, accidental oil spills, and almost all manufacturing industries release hazardous pollutants into the environment, including monocyclic hydrocarbons, polycyclic aromatic hydrocarbons (PAHs), chlorinated hydrocarbons, plastics, pesticides, TMs and their derivates (Varjani et al. 2018; Sharma et al. 2024). There is a growing interest in using extremophiles for biodegradation of pollutants and bioremediation (Napper and Thompson 2023), and studying adaptive mechanisms that allow them to survive in highly polluted environments (Haripriyan et al. 2022). Biodegradation refers to the natural breakdown of various compounds by living organisms, and bioremediation is the targeted application of organisms and their enzymes to clean up polluted environments. Both approaches often use microbial activity to degrade toxic substances into less harmful or harmless products and they are considered to be effective, safe and economically convenient methods for removing environmental contaminants (Dave and Das 2021). Figure 3 provides a schematic overview of the principal mechanisms employed by extremophiles in environmental biotechnology, including plastic biodegradation, bioleaching and detoxification of toxic metals, and biodegradation of PAHs under extreme environmental conditions. The figure summarizes representative extremophilic microorganisms, key degradation pathways, major enzymes and genes involved in pollutant degradation, as well as current limitations associated with these biotechnological processes. The following sections discuss the major applications of extremophiles in environmental biotechnology in detail.

### Biodegradation of plastics

The widespread use of plastics is closely linked to their favourable chemical and physical properties, including low weight, durability and relatively low production costs. Consequently, synthetic polymers such as polyethylene (PE), polypropylene (PP), polyvinyl chloride (PVC), polyethylene terephthalate (PET) and polystyrene (PS) have become essential materials in industries ranging from food packaging to advanced technologies. Since the beginning of large-scale plastic production in the 1950s (Napper and Thompson 2023), the continuous increase in plastic consumption has resulted in severe environmental pollution caused by inefficient recycling, uncontrolled accumulation and the growing presence of microplastics in diverse ecosystems (Ziani et al. 2023). Most synthetic plastics are highly resistant to natural degradation processes, and therefore, the development of environmentally sustainable strategies for their removal is necessary (Wu et al. 2024).

The biodegradation of plastics proceeds through four principal stages: biodeterioration, depolymerization, assimilation, and mineralization (Heris 2024). During biodeterioration, plastics are exposed to both microbial enzymatic activity and various abiotic factors. This phase involves physicochemical and mechanical alterations of polymer structures, often facilitated by the formation of microbial biofilms. Following these initial changes, depolymerization occurs (Liu et al. 2025). This step is driven primarily by extracellular and intracellular depolymerase enzymes that cleave long polymer chains into smaller intermediates such as oligomers, dimers, and monomers. These degradation products are typically water-soluble, allowing more efficient cellular uptake (Heris 2024). The subsequent assimilation stage takes place within the cytoplasm of microbial cells, where the absorbed intermediates are metabolized to generate energy, biomass, and primary or secondary metabolites. The final step of the biodegradation process is mineralization, during which microbial biomass and residual organic compounds are converted into less harmful end products, including CO₂, N₂, CH₄, water, and inorganic minerals (Atanasova et al. 2021). This environmentally friendly approach does not only reduce plastic accumulation but also lowers the associated waste management costs (Heris 2024).

Biodegradation efficiency depends on the composition of the microbial community, the ability of microorganisms to colonize plastic surfaces, and enzyme suitability for depolymerization (Pinar and Rodríguez-Couto 2024). Key enzymes such as depolymerases, lipases, esterases, cutinases, chitinases, xylanases, keratinases, and carboxylesterases play a vital role in the breakdown of plastics. These enzymes are produced by bacteria, including mainly genera *Arthrobacter* (class Actinomycetes), *Rhodococcus* (class Actinomycetes), *Bacillus (Bacillus subtilis* and *Bacillus amyloliquefaciens*, class Bacilli), *Pseudomonas* (*Pseudomonas putida*, class Gammaproteobacteria) and fungal genus *Aspergillus* (Sol et al. 2020). Although many microorganisms can degrade plastics (Jacquin et al. 2019), increasing focus is currently on the degradation of plastics by extremophilic microorganisms.

PE degradation is similar to lignin decomposition, with key roles played by extracellular enzymes such as laccase, lignin peroxidase, manganese peroxidase, and hydrolytic enzymes like amylase, lipase, and esterase. These enzymes disrupt polyethylene’s structure and cleave its carbon bonds, aiding its breakdown (Ndahebwa Muhonja et al. 2018). For example, manganese peroxidase from crust fungus *Phanerochaete chrysosporium* can reduce polyethylene’s tensile strength and hydrophobicity, accelerating its biodegradation (Pinar and Rodríguez-Couto 2024). Similarly, alkane hydroxylase genes (alkB) and laccase-associated pathways have been implicated in PE oxidation and depolymerization, leading to formation of lower-molecular-weight intermediates that are more accessible for microbial assimilation (Pinar and Rodríguez-Couto 2024).

In PET-degrading microorganisms, genes encoding PET hydrolases (PETase) and mono(2-hydroxyethyl) terephthalate hydrolases (MHETase) catalyze sequential hydrolysis of PET into terephthalic acid (TPA) and ethylene glycol (EG), which can subsequently enter central metabolic pathways (Palm et al. 2019). *Vibrio gazogenes* (class Gammaproteobacteria) is a promising marine halophilic bacterium capable of producing PETase (PET6 enzyme), which efficiently degrades polyethylene terephthalate under saline conditions (Weigert et al. 2022; Pinar and Rodríguez-Couto 2024). In several extremophilic microorganisms, expression of these enzymes associated with biodegradation of plastics is enhanced by biofilm formation and biosurfactant production, which improve plastic surface colonization and substrate bioavailability (Katnic and Gupta 2025). Thermophiles, acidophiles, and halophiles exhibit remarkable capabilities for degradation of plastics under extreme conditions (Atanasova et al. 2021). Psychrophilic bacteria, often associated with biofilm formation, can adhere to plastic surfaces and establish stable microbial communities. These biofilms shield microbes from harsh environmental conditions while enhancing metabolic activity, leading to more efficient degradation of plastics. Notable bacterial psychrophiles, which have the potency for degradation of plastics at temperatures only slightly higher than zero, include gammaproteobacterial genera *Shewanella* and *Psychrobacter* (Goswami et al. 2024).

Halophilic microorganisms play a crucial role in degrading plastics in marine environments. Marine halophiles capable of plastics decomposition include genera *Alcanivorax* and *Enterobacter* (class Gammaproteobacteria), *Burkholderia* and *Pandoraea* (class Betaproteobacteria), and *Marinobacter* (class Alphaproteobacteria) (Krumov et al. 2025; Alharbi et al. 2026). Thermophilic microorganisms have demonstrated effectiveness in breaking down synthetic polymers at elevated temperatures. For instance, *Bacillus pallidus* (class Bacilli) degrades nylon 12 at 60 °C (Tomita et al. 2002), while *Brevibacillus borstelensis* (class Bacilli) reduces polyethylene mass by 11% within 30 days at 50 °C (Hadad et al. 2005). Another notable thermophile, *Thermobifida fusca* (class Actinomycetes), degrades PET by up to 50% at 55 °C (Müller et al. 2005). Despite these findings, research on thermophilic biodegradation of plastics remains limited (Pinar and Rodríguez-Couto 2024).

The study of extremophiles and their extremozymes capable of degrading plastics opens up new strategies for sustainable plastics recycling and microplastics decomposition, offering a promising approach for reducing pollution by plastics (Weigert et al. 2022). Due to the increasing environmental crisis, further research into microbial biodegradation of plastics is essential. The discoveries of novel extremophilic microorganisms capable of degradation of plastics could significantly enhance environmentally friendly waste management.

### Bioleaching – microbial metal leaching

Similarly, like pollution by plastics, environmental contamination by TMs poses a significant and increasing threat that demands urgent attention. Major waste sources containing metals metals include fly ash (Cu, V, Ni), discarded batteries (Zn, Mn, Li, Ni, Cd), spent refinery catalysts (Al, Ni, Mo), and electronic waste (Au, Cu, La, Ag) (Adetunji and Erasmus 2024). While some metals (Fe, Cu, Zn, Mn, Ni, Co, Mo) are essential trace elements involved in numerous enzymatic and physiological processes, excessive exposure to these metals can result in cellular toxicity (Zhu et al. 2024). If not properly managed, TMs can severely harm ecosystems and human health, contributing to DNA damage and diseases such as cancer, heart failure, dermatitis, and congenital defects (Adetunji and Erasmus 2024).

Traditional methods for metal removal include pyrometallurgy and hydrometallurgy. Pyrometallurgy relies on high-temperature processes but is often restricted or banned due to high energy consumption, and the release of toxic gases such as dioxins and furans. Hydrometallurgy, on the other hand, involves leaching metals with chemical agents, which are often hazardous to the environment and generate large amounts of secondary waste requiring further disposal (Naseri et al. 2023). Due to the environmental and economic drawbacks of these methods, alternative approaches have been suggested in recent years, with bioleaching emerging as one of the most promising solutions (Dash et al. 2025). Bioleaching, the biological leaching of metals using microorganisms, is an environmentally friendly, cost-effective, and highly efficient technology. The process is based on the activity of bacteria or fungi that produce inorganic or organic acids, leading to the gradual dissolution of metal compounds (Biswal et al. 2018). Some studies suggest that bioleaching can be more effective than hydrometallurgy. For example, Biswal et al. (2018) tested the use of filamentous fungus *Aspergillus niger* (class Eurotiomycetes) for processing lithium-ion battery powder. The results demonstrated that this fungus effectively removed Li and Co from the mixture, with an efficiency comparable to or even higher than conventional hydrometallurgy. However, a key limitation of microbial metal leaching is that TMs, despite undergoing metabolic transformations, are often retained in the biomass. Therefore, additional processing steps are required for their removal from the biomass, for example, by magnetic separation techniques (Wibowo et al. 2025).

From the perspective of NGIB, extremophilic bacteria, particularly autotrophic acidophiles from the class Acidithiobacillia, such as *Acidithiobacillus prosperus*,* Acidithiobacillus caldus*,* Acidithiobacillus thiooxidans*,* Acidithiobacillus ferrooxidans*,* Acidithiobacillus concretivorus*,* Acidithiobacillus albertis*, as well as *Leptospirillum ferrooxidans* from the class Nitrospiria, have enormous potential for bioleaching (Biswal et al. 2018). These microorganisms can efficiently process various types of solid waste, including tannery sludge, carbide slag, spent refinery catalysts, mine tailings, low-grade ores, but also polychlorinated biphenyls (PCBs) (Naseri et al. 2023).

In addition to acidophiles, thermophilic archaea such as genera *Sulfolobus* and *Metallosphaera* (class Thermoprotei) play a crucial role in bioleaching (Coker 2016). Bacterium *Sulfobacillus thermosulfidooxidans* (class Clostridia) is capable of oxidizing sulphur and iron (Chen et al. 2023) and it has been intensively studied for its ability to extract metals from sulphide ores and act as a bioadsorbent of TMs (Cd²⁺, Cu²⁺, Zn²⁺, and Ni²⁺) from acidic solutions (Huang et al. 2020).

These bioleaching processes are of major industrial and environmental importance because they enable the recovery of valuable metals from low-grade ores and industrial waste materials that are often economically unsuitable for conventional metallurgical extraction. Biomining technologies may reduce energy consumption and decrease the environmental impact associated with pyrometallurgical and chemical extraction methods. Therefore, extremophile-based bioleaching is a promising strategy for sustainable resource recovery and circular economy approaches in the mining and metallurgical industries (Johnson 2014; Biswal et al. 2018), with high potential also for cleaning the areas polluted by TMs.

### Degradation of polycyclic aromatic hydrocarbons

Hydrocarbons, the main constituents of petroleum pollution, persist in the environment due to their strong hydrophobicity and low solubility in water. Polycyclic aromatic hydrocarbons (PAHs) are toxic, mutagenic and carcinogenic compounds, whose fate in ecosystems is influenced by abiotic and biotic factors, including volatilization, adsorption, and microbial degradation (Duran and Cravo-Laureau 2016). They originate from both natural and anthropogenic processes, and they pose serious concerns for aquatic life and human health. Marine contamination is caused by oil spills during transportation, exploration, extraction, and the discharge of industrial wastewater (Ron and Rosenberg 2014). Every year, approximately 1.7–8.8 million metric tons of hydrocarbons are released into the oceans due to oil spills, leading to severe ecological consequences (Ambaye et al. 2022). In addition to petroleum pollutants, PAHs include pesticides that contaminate nature and disrupt biodiversity. Excessive PAH contamination significantly threatens the survival of many animal species (Mahmood et al. 2016). Moreover, plastics and tobacco smoke also contain PAHs, and they also arise during petroleum and coal combustion (Swaminaathan et al. 2024).

Bioremediation is an environmentally friendly alternative to physical and chemical methods for the decomposition of PAHs. However, its efficiency is limited by the hydrophobicity of hydrocarbons, their bioavailability and environmental conditions, and it takes longer than traditional remediation methods. Nevertheless, the complete degradation of these contaminants is often accomplished (Koshlaf and Ball 2017). Several reports describe the biodegradation of hydrocarbons under normal conditions, but only a limited number of studies have focused on extremophilic environments (Arulazhagan et al. 2017). *Pseudomonas* (class Gammaproteobacteria), *Bacillus* and *Geobacillus* (class Bacilli), *Petrobacter* (class Betaproteobacteria), and *Clostridium* (class Clostridia) (Koul et al. 2021) are among the genera which play a key role in the degradation of petroleum compounds.

The microbial degradation of PAHs involves a series of enzymatic oxidation and ring-cleavage reactions that convert complex PAHs into less toxic intermediates. The initial and often rate-limiting step is typically catalyzed by oxygenases, particularly monooxygenases and dioxygenases, which introduce oxygen atoms into aromatic rings and generate cis-dihydrodiol intermediates. In many hydrocarbon-degrading bacteria, genes encoding ring-hydroxylating dioxygenases (RHDs), such as naphthalene 1,2-dioxygenase (nahA), play key roles in the degradation of low- and high-molecular-weight PAHs (Duran and Cravo-Laureau 2016; Koul et al. 2021). Subsequent enzymatic reactions involve dehydrogenases, hydrolases and catechol-cleavage dioxygenases, leading to the formation of central metabolic intermediates, such as catechol, protocatechuate and pyruvate, which can enter the tricarboxylic acid (TCA) cycle. In extremophilic microorganisms, these degradation pathways are often supported by specialized enzymes with enhanced stability and catalytic activity (Jamal and Pugazhendi 2018).

In acidic settings (pH < 4), several acidophilic or acidotolerant bacteria have been associated with hydrocarbon degradation, particularly members of Alphaproteobacteria such as genera *Acidocella* and *Acidosphaera* (Röling 2010). In contrast, hydrocarbon degradation under alkaline conditions is less frequently reported, although certain alkalitolerant bacteria, including representatives of Actinomycetes, such as the genus *Mycobacterium*, have also been shown to have this ability (Dudhagara and Dave 2018). The petrochemical industry often releases highly saline and alkaline wastewater containing petroleum hydrocarbons, which require thorough treatment before being discharged into the environment. Effective degradation of these pollutants requires a halo-alkalithermophilic microbial consortium (Chen et al. 2017). Research has focused on developing novel extremophilic bacterial consortia capable of degrading various petroleum hydrocarbons in refinery wastewater under integrated extreme conditions (Al-Mur et al. 2021). Various studies suggest that salt-tolerant consortia are beneficial for PAH-degradation (Jamal and Pugazhendi 2018). Several halophilic and halotolerant bacterial genera have been identified as important contributors to PAHs degradation in saline petroleum wastewater. Frequently reported genera include *Pseudomonas* and *Stenotrophomonas* (class Gammaproteobacteria), *Achromobacter* (class Betaproteobacteria), and *Mesorhizobium* and *Brucella* (class Alphaproteobacteria) (Zhang et al. 2015). The 2015 research project funded by the King Abdulaziz City for Science and Technology (KACST) (Arulazhagan et al. 2017) demonstrated that halophilic bacterial consortia, including *Ochrobactrum* (class Alphaproteobacteria) and *Pseudomonas* (class Gammaproteobacteria) as dominant genera, enriched with saline water degraded up to 86% of phenanthrene (1500 ppm) at NaCl concentration 200 g/L (Arulazhagan et al. 2017). Given the high salinity of petroleum wastewater, research into halophilic microorganisms is essential for effective bioremediation strategies removing PAHs.

Thermophilic bacilli, such as *Geobacillus stearothermophilus*, can degrade certain petroleum products as well, while *Bacillus thermoleovorans* is specifically capable of degrading naphthalene at 60 °C using metabolic pathways distinct from mesophilic species (Swaminaathan et al. 2024). The complete degradation of PAHs at 60 °C using a bacterial consortium from an oil well, consisting of various *Pseudomonas* species producing biosurfactants, has also been reported (Verma et al. 2020). Additionally, several psychrophilic bacteria, such as some *Bacillus* spp. (Kolsal et al. 2017), *Sphingomonas* spp. (class Alphaproteobacteria) (Al Farraj et al. 2021) and *Shewanella putrefaciens* (class Gammaproteobacteria) (Li et al. 2024), have been shown to exhibit significant PAH-degrading capabilities.

## Challenges and future perspectives

Despite the considerable potential of extremophiles in environmental biotechnology, several important challenges still limit their widespread industrial and environmental application. One major limitation is the difficulty associated with large-scale cultivation of many extremophilic microorganisms, as numerous species require highly specialized growth conditions that are difficult to reproduce, and 99% of microorganisms can not be cultivated even at the low scale level (Sysoev et al. 2021). Moreover, some extremophiles exhibit relatively slow growth rates and lower biomass yields compared with mesophilic microorganisms, which may reduce the usefulness for bioremediation purposes (Abdul Rehman et al. 2026). The effectiveness of bioremediation is also limited by low pollutant bioavailability and fluctuating environmental conditions, particularly in contaminated soils (Alori et al. 2022) and hypersaline wastewaters (Rezaei et al. 2025). Furthermore, introduced extremophilic strains or consortia may compete with native microbial communities, potentially affecting the long-term stability and efficiency of bioremediation systems in situ (Romantschuk et al. 2023).

From the perspective of NGIB, extremophiles represent attractive production platforms because their extreme cultivation conditions may reduce contamination by mesophilic microorganisms and simplify non-sterile industrial processes (Wang et al. 2024b). However, large-scale cultivation of extremophiles is an engineering challenge. Highly saline or extreme pH conditions may accelerate corrosion of bioreactor components and increase operational costs associated with specialized materials, process control and wastewater management (Zhu et al. 2020). Consequently, further advances in bioreactor engineering will be essential for the efficient implementation of extremophile-based biotechnological and bioremediation approaches.

Another major challenge concerns the identification and functional annotation of extremozymes (Mesbah 2022). Since most available enzyme databases are dominated by mesophilic sequences, homology-based annotation of extremophilic proteins remains difficult due to their distinct amino acid composition connected with structural adaptations (Mesbah 2022). Nevertheless, recent advances in metagenomics, bioinformatics, single-cell genomics and synthetic biology have enabled the discovery of novel extremozymes without the need for cultivation of extremophile species and the genetic modification techniques have facilitated transfer of extremophilic traits to more easily cultivable microbial hosts (Mesbah 2022; Rezaei et al. 2025). The improvements in bioreactor design, hand in hand with genomics and genetic engineering, can increase extremozyme yields and the utility of extremophiles in biotechnology (Abdul Rehman et al. 2026).

Future research should therefore focus on improving cultivation strategies, development of robust microbial consortia, optimization of bioreactor systems, and integration of omics technologies, synthetic biology and metabolic engineering approaches (Rezaei et al. 2025). Advanced metagenomic and transcriptomic analyses may further improve understanding of extremophile metabolism and facilitate discovery of novel extremozymes with enhanced catalytic properties (Abdul Rehman et al. 2026). In addition, more pilot-scale and field-scale studies, and their comparisons, are required to evaluate the long-term stability, ecological safety and economic feasibility of extremophile-based bioremediation technologies under real environmental conditions (Anabtawi et al. 2025). Collectively, these advances may significantly contribute to the development of sustainable and efficient biotechnological strategies for the remediation of polluted environments (Giovanella et al. 2020). The environmental release of engineered extremophiles or other genetically modified microorganisms containing their genes requires careful evaluation because horizontal gene transfer and ecological interactions may influence native microbial ecosystems under field conditions (French et al. 2020).

## Conclusion

Extremophiles represent a highly promising group of microorganisms for environmental biotechnology owing to their unique metabolic adaptations and ability to survive under extreme environmental conditions. Their applications in biodegradation of plastics, petroleum-derived pollutants and toxic metals highlight their considerable potential for sustainable bioremediation and resource recovery. In addition to their ecological significance, extremophilic microorganisms and their enzymes offer important opportunities for the development of robust industrial biotechnological processes capable of operating under harsh conditions lethal to most model microorganisms. Despite substantial progress in recent years, important challenges associated with cultivation, scalability and process optimization limit the broader industrial and bioremediation applications of extremophiles. Continuing advances in systems biology, synthetic biology, metabolic engineering and environmental genomics are expected to significantly improve understanding and practical utilization of extremophiles in the future. With further technological development and field validation, extremophile-driven biotechnologies will likely become important tools for sustainable environmental management and circular bioeconomy strategies. 

## Data Availability

No datasets were generated or analysed during the current study.
